# Supplementary material for: Single-spore germination analyses reveal that calcium released during Clostridioides difficile germination functions in a feedforward loop
Source: mSphere. 2023 Jun 20;8(4):e00005-23. doi: 10.1128/msphere.00005-23 (PMC10449524; doi:10.1128/msphere.00005-23)
Supplement: TABLE S1 [file msphere.00005-23-s0002.docx]

**Supplementary Table S1**: *C. difficile* and *E. coli* strains used in this study.

***C. difficile* strains**

| **Strain #** | **Strain name** | **Genotype or relevant features** | **Reference** |
| --- | --- | --- | --- |
| 756 | 630∆*erm*∆*pyrE* | *erm*-sensitive derivative of 630 with *pyrE* deleted | [57] |
| 846 | 630∆*erm*-*p* | *erm*-sensitive derivative of 630 with *pyrE* restored | [50] |
| 1165 | 630∆*erm*∆*pyrE ∆dpaAB* | 630∆*erm∆pyrE* with *dpaAB* deleted | This study |
| 2251 | 630∆*erm*∆*pyrE ∆3298* | 630∆*erm∆pyrE* with *3298* deleted | This study |
| 2302 | 630∆*erm ∆3298-p* | 630∆*erm∆3298* with the *pyrE* locus restored | This study |
| 2306 | 630∆*erm ∆3298/3298* | 630∆*erm∆3298* with *3298* in the *pyrE* locus | This study |
| 2342 | 630∆*erm*∆*pyrE ∆spoVV* | 630∆*erm∆pyrE* with *spoVV* deleted | This study |
| 2345 | 630∆*erm ∆spoVV-p* | 630∆*erm∆spoVV* with the *pyrE* locus restored | This study |
| 2348 | 630∆*erm ∆spoVV*/*spoVV* | 630∆*erm∆spoVV* with *spoVV* in the *pyrE* locus | This study |
| 2419 | 630∆*erm*∆*pyrE ∆dpaAB/dpaAB* | 630∆*erm∆dpaAB* with *dpaAB* in the *pyrE* locus | This study |
| 2422 | 630∆*erm ∆dpaAB-p* | 630∆*erm∆dpaAB* with the *pyrE* locus restored | This study |
| 2428 | 630∆*erm*∆*pyrE ∆spoVAC* | 630∆*erm∆pyrE* with *spoVAC* deleted | This study |
| 2478 | 630∆*erm ∆spoVAC-p* | 630∆*erm∆spoVAC* with the *pyrE* locus restored | This study |
| 2481 | 630∆*erm ∆spoVAC/spoVAC* | 630∆*erm∆spoVAC* with *spoVAC* in the *pyrE* locus | This study |

***E. coli* strains**

| **Strain #** | **Strain name** | **Bencling plasmid map with primers** | **Reference** |
| --- | --- | --- | --- |
| 41 | DH5𝛂 | F– Φ80*lacZ*ΔM15 Δ(*lacZYA-argF*) U169 *recA1 endA1 hsdR17* (rK^–^, mK^+^) *phoA supE44* λ– *thi-1 gyrA96 relA1* | D. Cameron |
| 531 | HB101/pRK24 | F- *mcrB mrr hsdS20*(rB^–^mB^–^) *recA13 leuB6 ara-13 proA2 lavYI galK2 xyl-6 mtl-1 rpsL20* carrying pRK24 | C. Ellermeier |
| 1662 | HB101 pMTL-YN1C | <https://benchling.com/s/seq-0D3gMlPu6s87CIf9NBpG?m=slm-7Ap66iMh5U2JMK8tjaIv> | [50] |
| 1774 | HB101 pMTL-YN3-∆*dpaAB* | <https://benchling.com/s/k2qggHG9?m=slm-TXM8Kf6zZIdH4K1v8wId> | This study |
| 2238 | HB101 pMTL-YN3-∆*3298* | <https://benchling.com/s/seq-cKBGMNi2WGfQ3QSQr4Nz?m=slm-lTUrvwor7sD7TI2lr1zr> | This study |
| 2270 | HB101 pMTL-YN1C-*3298* | <https://benchling.com/s/seq-vuAomxcBiW2hK5LMN0Ea?m=slm-1byO9icu1SAW4yyiQcJR> | This study |
| 2273 | HB101 pMTL-YN3-∆*spoVV* | <https://benchling.com/s/seq-FZbE3SdBx2ddO7DbuAzE?m=slm-F66HDrlSmNepDBcfLINF> | This study |
| 2274 | HB101 pMTL-YN3-∆*spoVAC* | <https://benchling.com/s/seq-ra8zuMjrUxgS7qgR0Gqj?m=slm-YAZ9V5xiMEJkO39q3KhJ> | This study |
| 2293 | HB101 pMTL-YN1C-*spoVV* | <https://benchling.com/s/seq-XPApELaGhs9V7XQ0XTzK?m=slm-NnMPBkBQgKHPjX2vqfEa> | This study |
| 2294 | HB101 pMTL-YN1C-*spoVAC* | <https://benchling.com/s/seq-UaJraVcjrBQzBjt7wCM8?m=slm-EJYIoA0byXg8qjmk4DQE> | This study |
| 2295 | HB101 pMTL-YN1C-*dpaAB* | <https://benchling.com/s/seq-IkHZt67NyYzYRilvjFZF?m=slm-4qIT6MUatmuQCEnymM4n> | This study |

**Plasmids**

| **Plasmids** | **Relevant features** |  | **Reference** |
| --- | --- | --- | --- |
| pMTL-YN3 | For cloning allelic exchange constructs to manipulate 630∆*erm*∆*pyrE* | | [57] |
| PMTL-YN1C | For cloning complementation constructs to integrate genes into *pyrE* locus | | [57] |
